# Wounds and Arteries, Their Treatment

**Published:** 1855-03

**Authors:** 


WOUNDS AND ARTERIES, THEIR TREATMENT. By MR. BUTCHER.—The direction given in works on surgery to cut down to a wounded artery, and to put a ligature above and below the seat of injury, is good in itself, but not always practicable. When the internal carotid artery is wounded by a piece of tobacco pipe thrust through the mouth, the common carotid must be the vessel tied. A similar necessity may exist even in wounds of the extremities, as has been shown by Mr. Butcher, F. R. C. S. I., Surgeon to Mercer's hospital. We find, in the *Dublin Quarterly Journal of Medical Science*, an account of a wound of the profunda artery; ligature of the femoral, below the margin of Poupart's ligament, by a transverse incision; arrest of the bleeding; death eleven hours afterwards. It was impossible to tie the artery above and below the wound, owing to its great depth from the surface; the main trunk was, therefore, secured by a ligature, the incision through the skin being made transversely, as previously practiced by Mr. Porter, in consequence of the necessity of putting on the ligature so close to the crural arch. The most interesting part of Mr. Butcher's communication is the narrative of those cases in which he commanded hæmorrhage from a large vessel by means of well-adapted pressure. A policeman was stabbed in the leg; severe arterial hæmorrhage, followed by faintness, ensued. Mr. Butcher concluded, from the nature of the accident, that the posterior tibial artery was wounded. Graduated pressure, by means of a roller and compresses (seven or eight pledgets over the situation of the wound) was carefully exerted; the foot was raised; a compress and roller were applied over the popliteal artery, and a dose of morphia was administered; the limb was kept steady by a splint. On the following day, the pressure was removed from the popliteal artery and applied to the femoral, by means of an aneurism compress on the groin. The case did well; and Mr. Butcher, in commenting upon it, remarks that, as a rule, the surgeon should not seek for a wounded artery unless it be bleeding. A case of wound of the ulnar artery above the wrist was successfully treated by compression of the wound and pressure over the brachial artery; and profuse hæmorrhage from the hand after excision of the index finger, with removal of the head of the metacarpal bone, was completely controlled by powerful flexure of the injured limb, together with gentle pressure over both the radial and the ulnar arteries.—*Medical Chirurgical Review.*

